# Mechanism and Treatment Strategy of Osteoporosis after Transplantation

**DOI:** 10.1155/2015/280164

**Published:** 2015-07-27

**Authors:** Lei Song, Xu-Biao Xie, Long-Kai Peng, Shao-Jie Yu, Ya-Ting Peng

**Affiliations:** ^1^Center of Organ Transplantation, Second Xiangya Hospital of Central South University, Changsha 410011, China; ^2^Department of Respiratory Medicine, Second Xiangya Hospital of Central South University, Changsha 410011, China

## Abstract

Osteoporosis (OP) has emerged as a frequent and devastating complication of organ solid transplantation process. Bone loss after organ transplant is related to adverse effects of immunosuppressants on bone remodeling and bone quality. Many factors contribute to the pathogenesis of OP in transplanted patients. Many mechanisms of OP have been deeply approached. Drugs for OP can be generally divided into “bone resorption inhibitors” and “bone formation accelerators,” the former hindering bone resorption by osteoclasts and the latter increasing bone formation by osteoblasts. Currently, bisphosphonates, which are bone resorption inhibitors drugs, are more commonly used clinically than others. Using the signaling pathway or implantation bone marrow stem cell provides a novel direction for the treatment of OP, especially OP after transplantation. This review addresses the mechanism of OP and its correlation with organ transplantation, lists prevention and management of bone loss in the transplant recipient, and discusses the recipients of different age and gender.

## 1. Introduction

Organ transplantation is at present the only effective way to treat the end-stage diseases. But, at the same time, it increases the risk of osteoporosis (OP) and osteoporotic fractures which would have a serious impact on survival and life quality both in children and in adults [1–6]. The preoperative or postoperative factors lead to OP as well as osteomalacia and fracture. Generally, bone damage in transplant patients undergoes four phases: firstly, development of end-stage organ disease before transplantation; secondly, exacerbation immediately after transplantation caused by high-dose immunosuppressive therapy and continuing homeostatic disturbances; thirdly, a phase of stabilization secondary to immunosuppressive dose reduction and reestablishment of microenvironment of bone; fourthly, the return of OP caused by failing graft function. In particular, OP after renal transplantation may thoroughly tend to pass through the process above [7]. Within the different areas of transplantation, the mechanism of OP after transplantation has made considerable progress. Nonetheless, the related drugs for OP after transplantation are limited and lack pertinence in clinical practice. Owing to complex and diverse pathogenesis, strategies in the treatment and management of transplant patients with OP need to be categorized. This review will systematically investigate the prevention and treatment of OP in organ failure patients with different surgical state and population and summarize the progression of OP in scientific research and clinic.

## 2. Mechanism of Osteoporosis and Its Correlation with Transplantation

OP is characterized by a reduction in bone quality and bone mineral density, which usually gets worse with age. In particular, during the bone remodeling, the imbalance between bone formation and resorption will cause bone loss, which influences architecture of bone and attenuates the whole bone strength. Bone remodeling, which is mediated by osteoclasts (OC) and osteoblasts (OB) activities, is continuous in the whole life [8]. With the further research on the mechanism of OP, the important role of the molecule composed of osteoprotegerin/receptor activator of nuclear factor-*κ*B ligand (OPG/RANKL) [9, 10] in bone remodeling is striking; up to now, OPG/RANKL acts as a vital coupling factor between OC and OB. OPG and RANKL, produced by osteoblasts or bone marrow stromal cells, inhibit osteoclast differentiation and bone resorption activity. In addition, there are other factors or regulators that can influence the differentiation of OC or OB. An advanced research [11] showed that two important factors, complement component 3a (C3a) and collagen triple helix repeat containing 1 (Cthrc1), establish a bridge between OC and OB. C3a is derived from mature osteoclasts (mOC) and stimulates osteoblastogenesis, while Cthrc1 is secreted from mature active OC (maOC) in the middle of bone resorption and stimulates OB differentiation. The signal transduction pathways between OC and OB have long been shown to exist. Recent studies have demonstrated that several new transcription factors or regulators, such as nuclear factor I-C (NFI-C) [12], omentin-1 [13], and Netrin-4 [14], play different regulating roles in osteoblast proliferation and differentiation; moreover, they could also be used as novel therapeutic approach for treating OP. Some special type of osteoporosis, such as glucocorticoid- (GC-) induced OP (GIO), had already become a hot spot. At the gene regulation level, microRNA-29a (miR-29a) protects against GC-induced disturbance of Wnt and Dkk-1 actions and improves osteoblasts differentiation and mineral acquisition [15]; this study emphatically indicated the detrimental effects of GC treatment in association with reduced miR-29a expression; when the miR-29a function is enhanced, the side effects of GC treatment on mineral acquisition and osteoclast resorption are alleviated, and also RANKL expression is reduced, while knockdown of miR-29a accelerated the process above. Also, microRNA-17/20a inhibits osteoclastogenesis and bone resorption through blocking of RANKL expression in GIO [16]. Accordingly, the gene can regulate the differentiation of OC and OB by signaling pathway; moreover, it can be used as an alternative tactic for alleviating GC-induced bone deterioration. On the cellular level, in a previous study, Aggarwal et al. [17] found that the human umbilical cord blood-derived CD34^+^ cells induced bone formation in a murine model of OP and also showed that CD34^+^ transplantation increased trabecular numbers and thickness and increased bone mineral density (BMD), thereby indicating induction of osteoblast in bone. Above these, a safe conclusion to be drawn is that the OPG/RANKL system is the most important regulating mechanism in bone remodeling, while the other signaling pathway has increasingly become the research hot spot.

**Figure 1:**
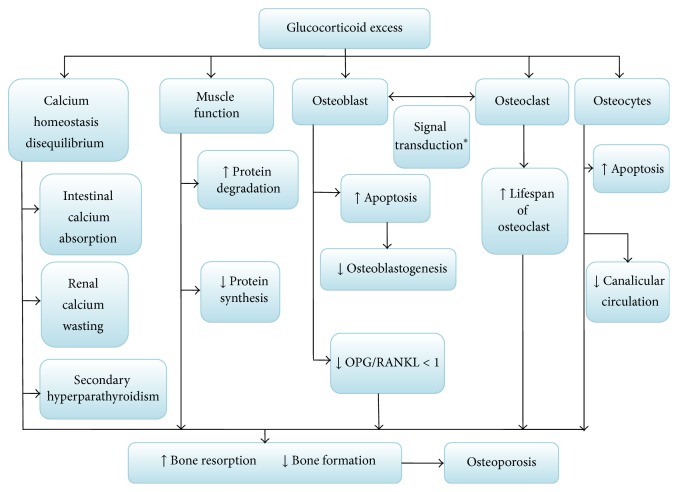
Effect of glucocorticoid excess on bone after transplantation. The atlas has indicated that glucocorticoid has direct and indirect pathway to mediate osteoporosis and inhibits bone formation after transplantation. The signal transduction pathway ^*∗*^: some factors establish a bridge between osteoblast and osteoclast, like complement component 3a (C3a) and collagen triple helix repeat containing 1 (Cthrc1), but there are few literatures after organ transplantation. The upward arrows show promoting effect; the downward arrows show lessening or inhibitory effects.

**Figure 2:**
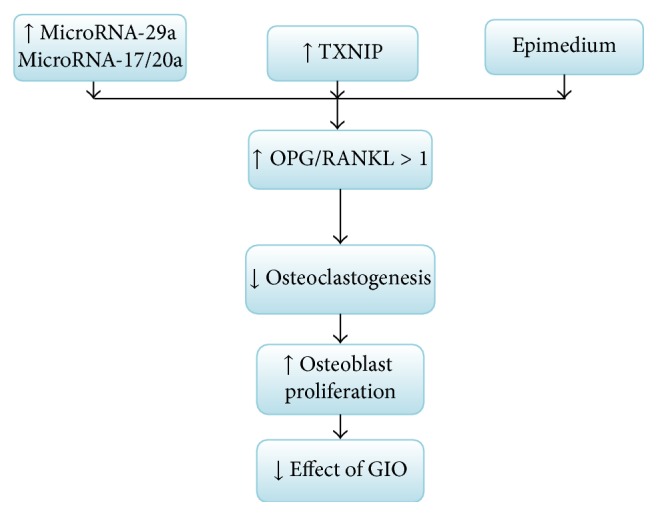
The protection mechanism against glucocorticoid-induced osteoporosis. MicroRNA-29a and microRNA-17/20a can inhibit osteoclastogenesis and promote osteoblast proliferation. Epimedium, which is the Chinese patent medicine, can antagonize the abnormal expressions of OPG and RANKL mRNA. The gene encoding TXNIP may increase the ratio of OPG/RANKL to downregulate osteoblast-mediated osteoclastogenesis. These potential protection mechanisms can prevent the progression of GIO and can provide a feasible and effective guidance to the treatment of osteoporosis after transplantation. The upward arrows show promoting effect; the downward arrows show lessening or inhibitory effects.

In the field of organ transplantation, OP is one of the major complications. The OPG/RANKL system is also involved in the pathogenesis of OP after transplantation. Many immunosuppressants directly or indirectly affect the reconstruction and absorption of bone throughout OPG/RANKL system. GC plays a critical role in the mechanisms of bone loss, such as reduced intestinal calcium absorption and renal calcium wasting and both may lead to a secondary hyperparathyroidism. Indeed, GC induces apoptosis of osteoblasts and osteocytes and prolongs lifespan of osteoclast, resulting in low bone mass and microarchitectural deterioration of bone tissue, which leads to severe OP. In this intricate process, van Staa [18] found that GC stimulates osteoclastogenesis by the regulation of OPG/RANKL, and one research has confirmed that an anti-RANKL antibody can protect the bone from loss in mouse model of GIO [19], revealing that OPG/RANKL is crucial for the induction of GIO. At the early period of posttransplantation, an excessive amount of GC must be administered in order to gain the immunosuppressive effect. With regard to GC excess, some researches confirmed that it was directly associated with osteoblast and osteocyte apoptosis in a transgenic mouse model of cell-targeted disruption of GC signaling [20, 21]. Hence, high-dose GC negatively affects osteoblast and osteocyte function. Several studies [22, 23] indicated that these actions include a decrease in the ratio of OPG/RANKL, which increased bone resorption and reduced bone formation, and also demonstrated that the Wnt signaling pathway may be involved in the GC-induced suppression of OPG. Obviously, the OPG/RANKL ratio controls the absorption of osteoclasts on bone; that is to say, the ratio <1 suggests a RANKL predominant activity and bone resorption, while an OPG/RANKL ratio >1 reveals OPG greater activity, and the bone protection process was predominant [24]. This conclusion has been authenticated in the bone marrow microenvironment after allogeneic hemopoietic stem cell transplantation [25]. Only when GCs bind to glucocorticoid receptors (GR) can they exert their functions [26] and then induce the latter conformational change; thus the activated GR can regulate gene expression in a negative way (transrepression), which causes their anti-inflammatory effect (also called immunosuppressive effect). GCs act primarily via the GR in bone cells to induce bone loss [27, 28]. In GIO model, GR in osteoblasts was sufficient to lead to GC-mediated bone loss, while GR in osteoclasts was insufficient. One study [29] confirmed that osteoclastogenesis could be enhanced in the initial phase of GC exposure. The enhanced osteoclastogenesis can decrease the ratio of OPG/RANKL. With regard to excess GC, it can suppress bone formation through inhibition of osteoblastic gene transcription [21]. Additionally, excess GC, through increased protein degradation and decreased protein synthesis, can also adversely affect muscle function; moreover, the riskiness of fragility fractures steadily increases by muscle weakness [30]. However, fortunately, OB can partly regulate the detrimental effects of GC; a frontier research [31] indicated that gene encoding TXNIP may increase the ratio of OPG/RANKL to disfavor OB-mediated osteoclastogenesis. Meanwhile, Epimedium [32], the Chinese patent medicine, can antagonize the abnormal expressions of OPG and RANKL mRNA in the GIO model; thereby it prevents the progression of GIO. Nevertheless, similar studies have not been reported in the GIO after transplantation. Hence, as will be readily seen, on the one hand, GC has direct and indirect pathway to mediate OP and inhibits bone formation (Figure 1); on the other hand, the protection mechanism of OB may play a considerable role in GIO, even treatment for GIO after organ transplantation (Figure 2).

**Figure 3:**
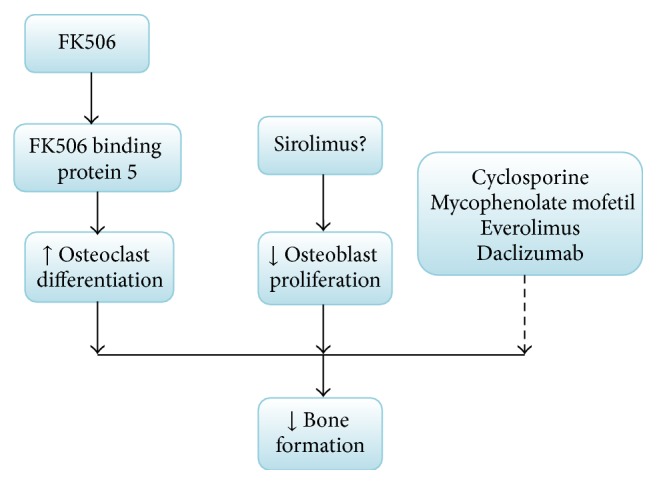
Effect of the nonglucocorticoid immunosuppressant on bone. FK506 binding protein 5 (FKBP5) messenger RNA (mRNA) can promote osteoclast differentiation, involved in glucocorticoid-induced osteoporosis; the function of sirolimus (SRL) on bone should be more discussed. The dashed arrows show that the other nonglucocorticoid immunosuppressants may not influence bone formation and bone mass or are still being evaluated for their skeletal effects.

The CI-based immunosuppression regimens, including cyclosporine (CsA) and tacrolimus (FK506), have been linked to OP in adult transplant recipients [33]. The murine experiment [34] suggests that FK506 binding protein 5 (FKBP5) messenger RNA (mRNA) in bone marrow can promote osteoclast differentiation by a mechanism distinct from NF-*κ*B activation and might play a role in GIO. Accordingly, FK506 has a negative effect on bone. On the contrary, CsA does not adversely affect bone metabolism or accelerate GIO [35]. The effects of the other immunosuppression regimens like mycophenolate mofetil, sirolimus (SRL), and everolimus on bone have a discrepancy. A recent in vitro study [36] suggests sirolimus might interfere with the proliferation and differentiation of osteoblasts, while a previous research [37] showed SRL was a “bone sparing immunosuppressant,” and it can increase the ratio of OPG/RANKL and has a potential to counteract deleterious GC effects on the bone. The function of SRL on bone should be more discussed. Everolimus [38] reduces cancellous bone loss in ovariectomized rats by decreasing osteoclast mediated bone resorption. Mycophenolate mofetil has no influence on bone formation and mass in clinical observations. Other new agents, such as daclizumab, are still being evaluated for their skeletal effects. But, no studies at present have confirmed that the effect of immunosuppressant on bone has a close correlation with OPG/RANKL, except for GC (Figure 3).

The OPG/RANKL system may be involved in the pathophysiological evolution of OP after transplantation. A study confirmed that the inseparable correlation between declined serum OPG levels and the relative bone loss has been observed in the early cardiac posttransplantation period, regardless of effective immunosuppressive therapy [39]. Fábrega et al. [40] revealed the same conclusion that OPG and receptor activator of RANKL may contribute to the development of OP late after orthotopic liver transplantation (OLT), and it is the activation of the immune system produced by the allograft that affects the release of both OPG and RANKL after liver transplantation. It seems that OPG/RANKL system is influenced by immune system in the organ transplantation. In addition to OPG, sclerostin, a circulating inhibitor of the Wnt-signaling pathway, has also received attention. Wnt-signaling pathway [41, 42] has a central role in regulating bone formation, and sclerostin inhibits bone formation. Thus, sclerostin is a protective factor in bone formation and Wnt signaling contributes to the development of OP. A research [43] concluded that the rapid reduction of elevated serum sclerostin levels one year after kidney transplantation parallels the improvement of renal function; the normalization of this hormone could contribute to improved bone health after renal transplantation. Hence, OPG/RANKL or Wnt signaling may be involved in the regulating mechanism of OP after transplantation, which is also influenced by immune system and affects the functions of graft.

All the mechanisms of OP and the relationship with transplantation have been discussed above, but the role of nonglucocorticoid (non-GC) immunosuppressants in posttransplantation bone disease is less well defined and needs more sophisticated research. Thus, a more comprehensive understanding of bone turnover and remodeling may lead to better therapeutic strategies to control OP in relevant diseases, especially after transplantation.

## 3. Drugs for the Treatment of Osteoporosis after Transplantation

### 3.1. Drug Therapy

At global clinical market, more than 20 kinds of drugs for OP have been developed, and they are broadly divided into 4 categories: calcium and vitamin D, antiresorptives, and bone formation stimulating and uncoupling regimens. With the increasing exploitation of new drugs, such as sclerostin inhibitors, bone formation stimulants, *α*V*β*3 integrin antagonists, cathepsin K inhibitor, calcium sensitive receptor antagonist, chloride channel inhibitors, nitrates, and so forth [44], the safety and effectiveness of these agents have attracted attention.

#### 3.1.1. Bisphosphonates

Within these new therapeutic agents above, some of them have been widely applied in clinical practice, whereas the research relating to the treatment of OP after transplantation is seldom involved. According to the PHARMAPROJECT database, bisphosphonates are the first drugs recommended for the treatment and prevention of postmenopausal OP and are still the hot spot in the OP treatment research. A few novel, longer acting, and more potent bisphosphonates like ibandronate, risedronate, and zoledronic acid may be given as infrequent, intermittent administration, which have been lately approved by US Food and Drug Administration [45]. A randomized case-control study [46] has shown that the use of alendronate sodium (Fosamax) (70 mg per week) for 14 months has a better curative effect without deteriorating renal function. Moreover, Fosamax significantly increased the bone mineral density (BMD) of hip in men more than in women. Abediazar and Nakhjavani [47] have shown that low-dose (30 mg) alendronate combined with vitamin D can increase the BMD immediately after renal transplantation. Also, the recent study [48] put forward the fact that a combination of vitamin D and bisphosphonate is the most effective protocol to improve BMD in renal transplantation recipients, but it also points out that patients who had persistent hyperparathyroidism could not use vitamin D and bisphosphonate only. Consequently, calcitriol (1,25-(OH)2D3) and alfacalcidol are also basic drugs that protect against bone loss. Among bisphosphonates, a research [49] had shown that pamidronate (90 mg, start 3 weeks after transplantation for 3 months) was comparable to alendronate in prevention of bone loss for the first six months after kidney transplantation. However, Torregrosa et al. [50] concluded that the administration of 60 mg pamidronate should be safe and has less adverse effects. Daily administration of bisphosphonates is limited by major gastrointestinal side effects [51]. A newer, orally administered bisphosphonate named risedronate can be well tolerated than others. Torregrosa et al. [52] also found that combination of risedronate (35 mg/week, oral administration) and vitamin D as well as calcium (800 IU cholecalciferol and 2500 mg of CaCO_3_/d) ameliorates BMD and bone pain in renal transplant recipients with established OP and also improves quality of life. A 1-year randomized, double-blind, placebo-controlled study [53] demonstrated that ibandronate (i.v. 3 mg, for 3 months) appeared to be safe and well tolerated for 12 months' treatment with early stable renal function (≤28 days following transplantation, GFR ≥ 30 mL/min); use of ibandronate alone did not show any benefit in preventing bone mineral density loss in the lumbar spine. On top of oral calcitriol 0.25 mg/day and calcium 500 mg b.i.d. is virtually maintaining BMD without any loss over 12 months after renal transplantation. In a multicenter, phase II, randomized open-label trial of intravenous zoledronic acid (ZA) (4 mg) to prevent BMD loss in adult recipients of allogeneic hematopoietic cell transplantation (alloHCT) with osteopenia before HCT, it had been confirmed that intermittent ZA is effective in preserving long-term bone health in adult alloHCT recipients at risk for OP [54].

#### 3.1.2. Recombinant Human Parathormone

Teriparatide is a recombinant human parathormone (PTH 1–34), which is an anabolic agent, currently only approved for the treatment of osteoporosis with high risk of fracture. A large sample study [55] has shown that patients who receive long-term GC treatment use teriparatide (20 *μ*g, once daily) to increase more bone mineral density than in those receiving alendronate. Nogueira et al. [56] strongly suggest that refractory hypocalcaemia after renal transplantation in patients with low PTH levels can be successfully treated with teriparatide; PTH analog therapy permits earlier suspension of intravenous calcium supplementation and reduces calcitriol requirements.

#### 3.1.3. Sclerostin Inhibitors

Denosumab, which is called anti-sclerostin antibody, is a RANKL inhibitor for treatment of postmenopausal OP. It can theoretically reduce osteoclastic resorption of trabecular structures, but currently the human data is as yet unproven [57]. Whether anti-sclerostin antibody treatment is efficacious to prevent bone loss after renal transplantation would need to be investigated. Antibodies targeting sclerostin increase bone growth in preclinical studies in osteoporotic monkeys [58]. In a phase I clinical study, a single dose of anti-sclerostin antibody (AMG 785, romosozumab) increased bone density in the hip and spine in postmenopausal women [59]. Hence, the inhibition of sclerostin might be a promising therapeutic strategy for the preservation of bone mass. However, the use of these drugs in the clinical routine of recipients is limited by their poor gastrointestinal tolerance, variable oral bioavailability, and long-term compliance, especially bisphosphonate. Moreover, the efficaciousness of anti-sclerostin antibody treatment to prevent bone loss after transplantation should be more investigated.

### 3.2. Bone Regenerative Therapy

At present, the regenerative bone therapy acts as source to treat osteoporosis, including embryonic stem (ES) cells and pluripotent stem (iPS) cells. ES cells are created from the inner cell mass of the blastocyst, an early-stage embryo, which has a high proliferative capacity in addition to pluripotency. A recent report confirmed the efficacious use of ES cells for the replacement of lost tissue, like bone [60]. Transplantation of allogenic ES cells also rises the risk of a rejection response in the recipient. However, iPS cells are largely free from ethical issues or the possibility of rejection by the immune system; they can differentiate into any cell type within the body. These induced pluripotent human stem cells were first successfully established from the human in 2007 [61]. A research had shown that transplantation of allogenic adipose-derived stromal cells (ASCs) can restore the BMD and bone histomorphometric properties of rats with glucocorticoid-induced OP (GIOP) and may serve as a potential treatment for GIOP [62]. Another study [63] also supports the use of ASCs as an autologous cell-based approach for the treatment of osteoporosis. Nevertheless, many ethical and technological issues should be more discussed. Not all of the bone regenerative therapies can treat osteoporosis after transplantation and the relative data are still absent. These researches above are anticipated to be extremely useful for the development of new therapeutic strategies, where previous strategies have failed.

## 4. Management of Immunosuppressive-Induced Osteoporosis

According to its characteristics of immunosuppressive-induced OP, the treatment of OP after transplantation is not only by using therapeutic drugs, but also by adjusting the dosage of immunosuppressive drugs. Numerous studies [64, 65] have also demonstrated that GC is a major contributor to bone loss after transplantation, especially the rapid bone loss that occurs in the first 6–12 months. Therefore, in the first years after transplantation, GC reduction or complete avoidance will be helpful to these patients. But, a randomized controlled trial [66] has shown that GC withdrawal, when carried out weeks to months after renal transplantation, is correlated with an increased risk of acute rejection. Hence, the current Improving Global Outcomes (KDIGO) guidelines [67] do not currently recommend GC withdrawal and avoidance as a routine course of action. So GC therapy should be given at the lowest possible therapeutic window in order to avoid acute rejection and delay the progression of OP. However, the specific data of GC avoidance or withdrawal protocol are conflicting clinically; one retrospective study [68] showed that all liver transplant patients who received GC >3500 mg in the first year have a much higher risk of bone disease than the group of GC <3500 mg and that female patients were worse than the male patients. Another trial [69] comparing early (7 days) GC cessation versus long-term, low-dose (5 mg/d after 6 months of transplant) GC therapy in 386 renal transplant patients showed that there is no discrepancy in the rate of bone loss at 5 years' follow-up. Moreover, for nonrenal transplant patients, there is a lack of evidence supporting GC avoidance or withdrawal protocol and there is no agreement on an ideal protocol. The other immunosuppressants are not proven by experiments that change the dosage of them for treating OP. So, no prescription modifying the immunosuppressive regimens, except for GC, has been significantly influenced in bone, and there is no good clinical evidence for choosing calcineurin inhibitors or other non-GC immunosuppressants to deal with OP.

## 5. Prevention and Management of Pretransplantation

In order to reduce the OP after transplantation, attention to comprehensive and rigorous preoperative prevention programs should be paid. Most patients undergoing transplantation will have preexisting bone disease, such as renal osteodystrophy, chronic kidney disease-mineral and bone disorder (CKD-MBD), osteitis fibrosa, and chronic obstructive pulmonary disease (COPD). There are many common factors causing the bone disease, which are persistent hyperparathyroidism (PTH), diabetes mellitus, water electrolyte disorder in dialysis, malnutrition, and so on, as well as anxiety, smoking, drinking, obesity, lack of sun exposure, age at menopause (women), and number of falls which became independent risk factors. One study has indicated that low vitamin D levels and bone disease are common among patients with end-stage liver disease awaiting liver transplantation [70]. To control these risk factors above, for example, treating primary bone disease and hyperparathyroidism, controlling blood sugar can effectively reduce the incidence of the osteoporosis and associated bone disease of renal transplant recipients and will extend their lifetime. Tseng et al. [71] found that a significant BMD decreasing was also found in the group of CKD stage ≥III, especially in women, and concluded that osteoporosis screening is necessary in patients with poor renal function. Dorn et al. [72] pointed out that the adolescent smokers are at higher risk for less than optimal bone accrual. Even in the absence of diagnosable depression, depressive symptoms may influence adolescent bone accrual. Physical activity should be encouraged during aging to reduce skeletal structural decay [73]. It can be safely concluded that lifestyle modification including healthy dietary practices and regular exercise, cigarette cessation, and avoiding moderate alcohol intake should be necessary. Vitamin supplementation, particularly vitamin D, should be considered to enhance diet based on patient's need.

## 6. Osteoporosis Prevention and Management of Posttransplantation

### 6.1. Paediatric

Transplantation may lead to secondary OP in children. In paediatric renal recipients, preexisting renal osteodystrophy at the time of kidney transplantation, GC treatment, and long-term graft function can be three major contributing factors [74]. A descriptive study [75] on bone histomorphometric findings pointed out that bone quality (i.e., abnormal turnover rate, thin trabeculae) rather than the actual loss of trabecular bone might account for the increased fracture risk in pediatric recipients; in addition, children with a higher present GC dose (≥3 mg/day) had significantly lower osteoclast (OC S/BS) (*P* = 0.018) and osteoid maturation time (Omt, *P* = 0.028) than children with the lower GC dose in this study. Recently, bone biopsy with tetracycline labeling and histomorphometry analysis is still the gold standard in assessing bone quality [76]. However, invasive examinations are not applied to children and noninvasive measures like peripheral quantitative computed tomography (pQCT) are not widely available. Hence, currently, it is recommended that PTH levels should be kept within the range appropriate for the CKD stage. Both native and active vitamin D are used to suppress PTH levels in CKD patients. Native vitamin D should be served as a first-line therapy in patients showing vitamin D insufficiency or deficiency (<30 ng/mL), while active vitamin D should be served as a second-line therapy. Accordingly, paediatric transplant patients should be given optimal nutrition, optimal treatment with vitamin D and calcium, and low dosage of steroids. And then regular physical activity is helpful for improving muscle and bone strength in children. Some studies [77] have indicated that GC withdrawal and recombinant human growth hormone (GH) therapy are helpful for attaining adult height. However, use of GH to treat OP of paediatric renal transplant patients is not yet common. El-Husseini et al. [78] had demonstrated that treatment of established bone loss with alendronate (5 mg/d, oral) is effective in young individuals even after the period of most rapid bone loss has already occurred and also indicated efficacy of intranasal calcitonin (200 IU/day) in the treatment of bone loss in young renal transplant recipients compared to the control group. But, the efficacy and safety of these drugs must be further proven in adequately designed clinical trials.

### 6.2. Women

Bone loss, especially in women, has been a concern with the long-term use of glucocorticoids and has been one of the driving forces behind steroid minimization and steroid withdrawal protocols. In addition, Brandenburg et al. [79] have confirmed that low estradiol and high luteotropic hormone (LH) levels correlated with the extent of annual BMD loss (*P* < 0.05) in postmenopausal renal transplant women; the lumbar T-scores reduced in the very late period after renal transplantation. Circulating sex hormones influence lumbar BMD. Estrogen supplements have a certain effect, but the side effects should be considered. Toro et al. [80] pointed out that OP was more frequent among female than male patients. The incidence rate of osteoporosis was higher among postmenopausal than premenopausal patients (50% versus 16.1%). In premenopausal women there was a negative correlation between the BMD of the vertebral column and PTH (*P* < 0.024). A cross-sectional study [81] has shown that the decrease of BMD during the menopause is associated with follicle-stimulating hormone (FSH) and luteinizing hormone (LH) levels, rather than estradiol (E_2_) in Chinese women. A prospective study on the mechanism of postmenopausal women OP indicated that the expression of ER-a36 in bone is positively associated with BMD and negatively associated with serum levels of the bone biochemical marker osteocalcin, and it mediates a bone-sparing effect of the low level of E_2_ in postmenopausal women [82]. Another study by Opelz and Döhler [83] corroborated the fact that the posttransplant fracture risk was increased for women, especially for women over 60 years of age who had a 5-fold increased risk of hip fracture. Hence, according to the characteristics of osteoporosis of female transplantation recipients, selecting the appropriate treatment may be important. At present, bisphosphonate therapy is a conventional method, yet there are many side effects. Dietary counseling to encourage all patients who begin receiving either oral or intravenous injection bisphosphonate therapy should have adequate calcium and vitamin D intake. A daily intake of 1200 mg of calcium is recommended for all women with osteoporosis, but high doses of calcium supplementation may cause increased kidney stone formation [84]. A research suggested that patients, over the age of 50 years, can receive oral administration of vitamin D in 800 IU, including supplements if necessary [85]. Current OP therapies have significant drawbacks; Joshua et al. [86] put forward the fact that cyclic GMP- (cGMP-) elevating agents may have bone-protective effects through nitric oxide/cGMP/protein kinase G (NO/cGMP/PKG) pathway. Accordingly, this provided a concept that soluble guanylate cyclase may act as a novel class of drugs, anabolic treatment strategy for postmenopausal OP. However, there are few clinical studies involving postmenopausal women who had undergone transplantation. Many new drugs cannot be used for clinical purposes, which should be more discussed.

### 6.3. Elderly People

Currently, although organ transplantation has already extended life expectation in older age groups, elderly renal recipients (defined as patients above 65 years old) need more consideration, in terms of not only selecting and waiting time, but also laying emphasis on their posttransplant and long-term care. Older transplant recipients had worse outcomes than younger recipients [87]. OP is a major concern during the whole life of transplant recipients [88]. So, the management of elderly recipients should be rigorously handled. Consequently, Mallet et al. [89] pointed out the impact of polypharmacy in general and its side effect on mortality and morbidity especially in aged patients. Hence, immunosuppressants have to be adapted to avoid both rejections and adverse effects. On the other hand, older transplant patients seem to have lower incidences of acute rejection episodes than younger patients.

Before transplantation, in elderly patients, there are numerous physiological conditions, such as reduced biomechanical strength, muscle fiber atrophy, calcium intake insufficiency, and vitamin D deficiency. All of which may determine a more complex bone metabolism alteration in elderly patients than in the young. With the research on the mechanism of senile OP, Leucht et al. [90] found that human bone marrow loses its osteogenic potential with age and aged bone grafts show a dramatic reduction in Wnt gene expression and Wnt responsiveness. This provided a new strategy for the treatment of skeletal injuries which is packaging Wnt protein into lipoparticles. Then after transplantation, using glucocorticoids (GCs) will accelerate bone loss, so older people are more likely to experience fracture. That is to say, GCs should be reduced to the appropriately lowest dose. Ahmadpoor et al. [91] showed that a hip or spine Z score of 1 or less had relationship to the total dosage of prednisolone (*P* < 0.001). The other drugs, such as bisphosphonate, had already been used clinically, but the efficacy and side effects have not been systematically and comprehensively evaluated; the side effect of these drugs on the senile people is more prominent than on young people. Although the new mechanism of action of drugs had already made progress, just a few researches had probed into treating the elderly recipients who had undergone transplantation.

## 7. Conclusion

Osteoporosis following transplantation is an intractable and intricate task; it increases mortality and decreases quality of life. Not only OP after surgery, but also preexisting osteoporosis should be paid more attention, and a healthy lifestyle is necessary. Reasonable and feasible individual treatment program can help improve efficiency.

There have been increasing numbers of studies highlighting the potential benefits of targeting signaling pathway or bone marrow stem cell therapy in OP, especially OPG/RANKL system. But the treatment strategy tailored to clinical use has not been implemented yet. At present, it seems that combination therapy with vitamin D and bisphosphonates and calcitriol (25-OHD) or alfacalcidol and right dose of glucocorticoids was the most effective and efficient regimen to improve BMD of these patients. More studies on animals and further translation to clinical practice should be done to explore more novel mechanisms that could open up new avenues for the treatment of these disorders.
